# Latitudinal variation in survival and immature development of *Ceratitis capitata* populations reared in two key overwintering hosts

**DOI:** 10.1038/s41598-023-50587-2

**Published:** 2024-01-03

**Authors:** Georgia D. Papadogiorgou, Antonis G. Papadopoulos, Cleopatra A. Moraiti, Eleni Verykouki, Nikos T. Papadopoulos

**Affiliations:** Department of Agriculture, Crop Production and Rural Environment, School of Agricultural Sciences, University of Thessaly, Vólos, Greece

**Keywords:** Invasive species, Population dynamics, Invasive species, Population dynamics

## Abstract

*Ceratitis capitata*, a major agricultural pest, is currently expanding its geographic distribution to northern, temperate areas of Europe. Its seasonal biology and invasion success depend on temperature, humidity and host availability. In coastal warmer Mediterranean regions and cooler temperature areas, bitter oranges and apples serve as overwintering hosts during the larval stage. We assessed the overwintering capacity of *C. capitata* populations obtained from different areas of the northern hemisphere by studying the survival and development rates of immature stages in both fruits under laboratory conditions. Eggs from each population were artificially inserted in the flesh of the two hosts and kept at 15, 20, or 25 °C until pupation and adult emergence. Climatic analysis of the area of the population origin showed combined effects of latitude, host and macroclimatic variables on immature survival and development rates. Egg to adult survival rates and developmental duration were longer in apples than in bitter oranges. For populations originated from southern-warmer areas, egg to adult developmental duration was prolonged and adult emergence reduced at 15 °C compared to those populations obtained from northern regions. Our findings reveal varying plastic responses of medfly populations to different overwintering hosts and temperatures highlighting the differential overwintering potential as larvae within fruits. This study contributes towards better understanding the medfly invasion dynamics in temperate areas of Northern Europe and other parts of the globe with similar climates.

## Introduction

In recent decades, the Earth’s near-surface temperature has substantially increased. Determining whether this global warming is relevant with human activities or is due to natural causes is crucial^1^. Climate change (e.g., temperature, relative humidity, winds and solar radiation) can affect not only the distribution but also the abundance of insects^2,3^. Invasive species refer to introduced species able to affect negatively environment, human activities and human health^4^. The ability of species to adapt to variable environments and environmental stress resistance determine persistence and abundance in specific habitats and regulate invasion dynamics^5^. The increase of global temperature is expected to favor invasive species over indigenous ones, as tropical and subtropical insects expand their geographic range into temperate areas^6^. In this context, the ability of insects to survive in climatic conditions different from those prevailing in the native range, is likely to contribute to their invasion potential^7^.

The Mediterranean fruit fly (medfly), *Ceratitis capitata* (Wiedemann), is a major agricultural pest of huge economic importance for fresh fruit production globally^8^. It holds an impressive record of successful invasion events, spreading in less than 200 years from its ancestral habitats, in the eastern parts of sub-Saharan Africa, to almost all temperate and tropical areas of the globe^9^. In recent years, either due to climate change or local adaptation, *C. capitata* is detected in cooler more temperate areas of Europe, threatening the deciduous fruit production^10^. Climate change and especially warmer winters, are suggested as driving environmental factors that can expand the geographic range of this pest in more temperate areas of Europe^10^. On the other hand, biological and physiological traits such as (a) thermal plasticity of insects under hot and cold conditions, (b) strong dispersal ability and (c) quick adaptation to new environments seem to enable medfly persistence and thriving in new environments^6^.

Although, the biology and ecology of *C. capitata* has been extensively studied, the extent to which physiological and life history traits facilitate its introduction to new environments, remains poorly understood^5^. Furthermore, interpopulation and genotypic variation and how this contribute to *C. capitata* invasions is unclear^11^. Geographic diversity of genotypes and phenotypes of medfly populations can emerge from adaptation to novel, invaded habitats^12^. Indeed, latitudinal clines in medfly populations have been shaped by natural selection in novel environments as it is proposed by Kourti and Hatzopoulos^13^. Diamantidis and colleagues^14,15^, following a common garden experimental approach, demonstrated that medfly biotypes originating from different geographic areas, express phenotypic variation in life history traits. Females from biotypes obtained from temperate areas outlived those originating from tropical ones^14^. Long lived individuals acquire an advantage, considering that they can bridge the absence of host fruits in the wild observed in cooler temperate areas. Hence, *C. capitata* biotypes that have colonized areas with different ecological characteristics differ in a range of biological characteristics that regard, both immature and adult stages^14^.

The distribution and abundance of *C. capitata* depends on several abiotic (e.g. temperature, rainfall, relative humidity, light intensity) and biotic factors (e.g. host plants, predators, parasitoids)^16^. Seasonal occurrence of the medfly population is related to ambient temperature, host availability and the overwintering capacity, especially in marginal for its existence environments^17–19^. Some hosts are highly favorable (e.g. species such as bitter oranges) for the immature development and adult performance, while others such as apples are not^20^. In temperate areas the abundance of different hosts (e.g. stone and pome fruits, figs etc.) leads to population growth, during the autumn^16,19^. These populations may infest late ripening hosts such as apples that may serve as an overwintering host as well in cooler areas such as northern Greece^21^. Prolongation of larval development inside infested apples seems to be the overwintering mechanism in cooler temperate areas, that assures the survival of a small number of individuals each winter to regenerate the entire population in spring and early summer. Furthermore, longer larvae developmental period enables medfly to accumulate the required nutrients from poor diets (e.g. apples)^22^.

Winter survival of medfly is higher in coastal areas, where citrus fruits are cultivated^18^. In those areas, during the cool period of the year, when average temperatures fall below 14–15 °C, *C. capitata* populations survive as larvae inside the fruits, as pupae in soil, or as adults. Hence, during the growing season (May to July), medfly population densities increase and then fluctuate until the end of autumn, when the number of flying adults is reduced, due to temperature drop^23^. Consequently, the overwintering capacity of invading medfly populations determine establishment in colder more temperate areas^24^.

A substantial amount of work has been conducted in recent years studying how demographic responses are affected by different temperatures (constant and fluctuating) and the ability of *C. capitata* to develop in a long list of potential host fruits^25–29^. Dionysopoulou and colleagues^16^, studied the response of immature stages of different medfly populations (laboratory adapted and wildish ones) to different host fruits (apples and bitter oranges). The findings showed that both larva-to-pupa and larva-to-adult survival exhibited comparable patterns, but significant variations were observed among the four medfly populations, two host fruits, and different temperature conditions. Further, larval developmental time and pupation rates were higher for larvae implanted in apples when compared to bitter oranges. Regarding the effect of dietary components on larval of *C. capitata*, Nash and Chapman^30^, demonstrated that larvae reared on high protein diets compared to those reared on low protein diets expressed higher survival rates and shorter developmental duration. The ability of medfly to adapt to different larval diets is a complex process that involves both genetic and environmental factors^31^. In addition, plasticity to environmental stress, expressed as extension of immatures developmental period may vary among *C. capitata* populations originating from different geographic regions and is crucial for its invasion success^31^. To the best of our knowledge there are no studies examining whether geographically isolated populations express adaptive plasticity in overwintering hosts and temperatures.

Environmental variability across different latitudes may be related with adaptive and/or plastic responses. However, there is currently limited research that delves deeply into this relationship^32,33^. The aim of the current study was to explore the performance of different medfly populations, spanning a latitudinal range of about 13°, to two key overwintering hosts under a range of temperature conditions. Following a common garden experimental approach, we used medflies from populations located in environmentally diverse habitats, to assure that are representative of the climatic variations observed in *C. capitata* habitats across the Northern Hemisphere’s temperate zone. To quantify the local climatic variability, we conducted a principal component analysis, on the primary bioclimatic factors of temperature and precipitation as described in Poikela and colleagues^34^. To investigate the complex nature of overwintering ability and distinguish whether latitudinal variations have evolved, the impact of both local climatic conditions (determined by bioclimatic variables) and latitude (a proxy for photoperiod) were tested. Understanding of how these attributes facilitate invasion, generates important data for predictive population modeling to estimate range expansion of *C. capitata* into cooler more temperate areas of Europe.

## Results

### Survival rates

**Figure 1:**
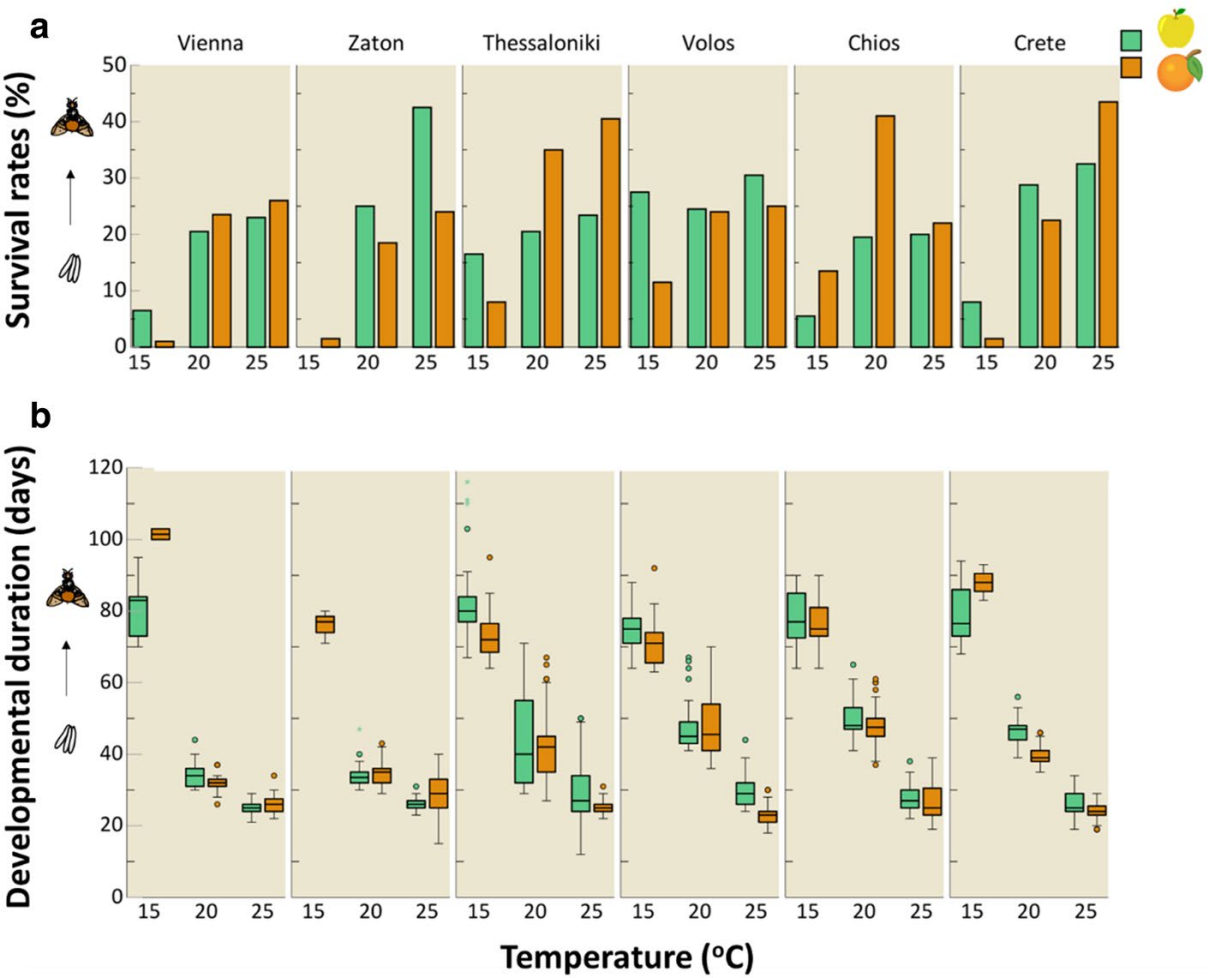
Effect of temperature on (**a**) survival rates from egg to adult (the length of each bar corresponds to the survival rates of each population, reared on the two hosts under different constant temperatures), and (**b**) developmental duration from egg to adult of different *C. capitata* populations, in two different overwintering hosts (apples and bitter oranges). Boxplots include the median, the 1st and the 3rd quartile. Whiskers indicate the lowest-highest value inside the interval defined by ± the 1.5-fold interquartile range from the 1st/3rd quartile.

Egg to adult survival rates of all tested populations, in both host fruits held under the three constant temperatures (15, 20, 25 °C) are given in Fig. 1a. With exception of sex all the tested factors had a significant effect on egg to adult survival rates (Table 1). Survival rates varied among populations, and this variation differed depending on the host fruit. Some populations performed better when reared in apples (Zaton, Volos, *P* < 0.001, respectively), while others performed better when reared in bitter oranges (Thessaloniki, Crete, *P* < 0.001, respectively) (Fig. 1a) (Supplementary Table S1). Furthermore, survival rates were influenced by the interaction between host and temperature (P < 0.001) (Table 1). In apples, an increase of temperature led to decreased survival compared to bitter oranges (Supplementary Table S2). Generally, at lower temperatures (15 °C), survival rates were higher when immatures were reared in apples, whereas at higher temperatures survival rates were higher in bitter oranges regardless of the tested population. The impact of temperature on survival rates varied among populations (Table 1). In most cases, an increase in temperature resulted in higher survival rates, except for immatures from Chios, where survival rates were higher at 20 °C compared to 25 °C (Fig. 1a). Finally, survival rates of the Crete and Zaton populations followed a similar pattern with temperature increase (*P* = 0.069) (Supplementary Table S2).

**Table 1:** Results of the linear models testing the effects of host, population, sex and temperature, on egg to adult emergence rates of *C. capitata* immature stages.

Variables in the model	Wald *x*^2^	df	*P*
Intercept	96.325	1	< 0.001
Host	89.707	1	< 0.001
Population	441.545	5	< 0.001
Sex	0.412	1	0.521
Temperature	1666.054	1	< 0.001
Host * population	1791.625	5	< 0.001
Host * sex	0.629	1	0.428
Host * temperature	124.210	1	< 0.001
Population * sex	0.253	5	0.998
Population * temperature	531.052	5	< 0.001
Sex * temperature	0.406	1	0.524

Details on the effect of temperature on survival rates of egg to pupa, and pupa to adult of different *C. capitata* populations, in two different overwintering hosts (apples and bitter oranges) are given in Appendix 1 (Supplementary Fig. S2, and Supplementary Tables S6 and S7), respectively.

### Developmental duration

Egg to adult developmental duration of the six medfly populations, in both apples and bitter oranges held under the three constant temperatures (15, 20 and 25 °C) is given in Fig. 1b. The results regarding the effect of host, population, sex, and temperature along with their interactions, are given in Table 2. Similar to survival rates, developmental duration among populations varied between the two hosts (Supplementary Table S3). Immatures obtained from Vienna (*P* = 0.017), Thessaloniki (*P* < 0.001), Volos (*P* < 0.001) and Crete (*P* < 0.001) had longest developmental duration when reared in apples. On the contrary, Zaton had longer developmental duration when reared in bitter oranges (*P* = 0.003), while the developmental duration in Chios was similar in the two hosts (*P* = 0.071). Notably, developmental duration varied between the two hosts, depending on the different temperature under which immatures had developed (*P* = 0.002) (Table 2). In apples the drop of temperature increased the developmental duration compared to bitter oranges regardless of the tested population and temperature (Supplementary Table S4). The interaction between temperature and population, had also a significant effect on immatures developmental duration. Generally, at low temperatures (15 °C), developmental duration was longer compared to high temperatures (25 °C). Temperature increase differentially reduced developmental duration of immatures in the geographically distant populations considered in the current study. However, temperature affected developmental duration of immatures in a similar manner in Crete (baseline), Thessaloniki (*P* = 0.796) and Volos (*P* = 0.930) populations, while developmental duration marginally differed between Chios and Crete (*P* = 0.049) (Supplementary Table S4). The developmental duration of male and female immatures significantly varied among populations (*P* = 0.026). Males obtained from Volos (*P* = 0.020) and Chios (*P* < 0.001), expressed longer developmental duration compared to females (Supplementary Fig. S1, Supplementary Table S5), while in other populations developmental duration was similar between females and males (*P* > 0.05).

**Table 2:** Results of the linear models testing the effects of host, population, sex and temperature, on egg to adult developmental duration of *C. capitata* immature stages.

Variables in the model	Wald *x*^2^	df	*P*
Intercept	9695.065	1	< 0.001
Host	16.152	1	< 0.001
Population	261.191	5	< 0.001
Sex	0.903	1	0.342
Temperature	4927.787	1	< 0.001
Host * population	52.624	5	< 0.001
Host * sex	0.532	1	0.466
Host * temperature	9.239	1	0.002
Population * sex	12.717	5	0.026
Population * temperature	215.274	5	< 0.001
Sex * temperature	0.343	1	0.558

Details regarding the effect of temperature on developmental duration of egg to pupa and pupa to adult of different *C. capitata* populations, in two different overwintering hosts (apples and bitter oranges) are given in Appendix 2 (Supplementary Fig. S3, and Supplementary Tables S8 and S9).

### Effects of macroclimatic variability of the sites of population origin

The temperature-precipitation background of the six population origin sites was characterized by principal component analysis (PCA) on 19 bioclimatic variables (see Supplementary Tables S10 and S11). The first two principal components (PCs) explained the 83.3% of the total variation (see Supplementary Table S12).

**Figure 2:**
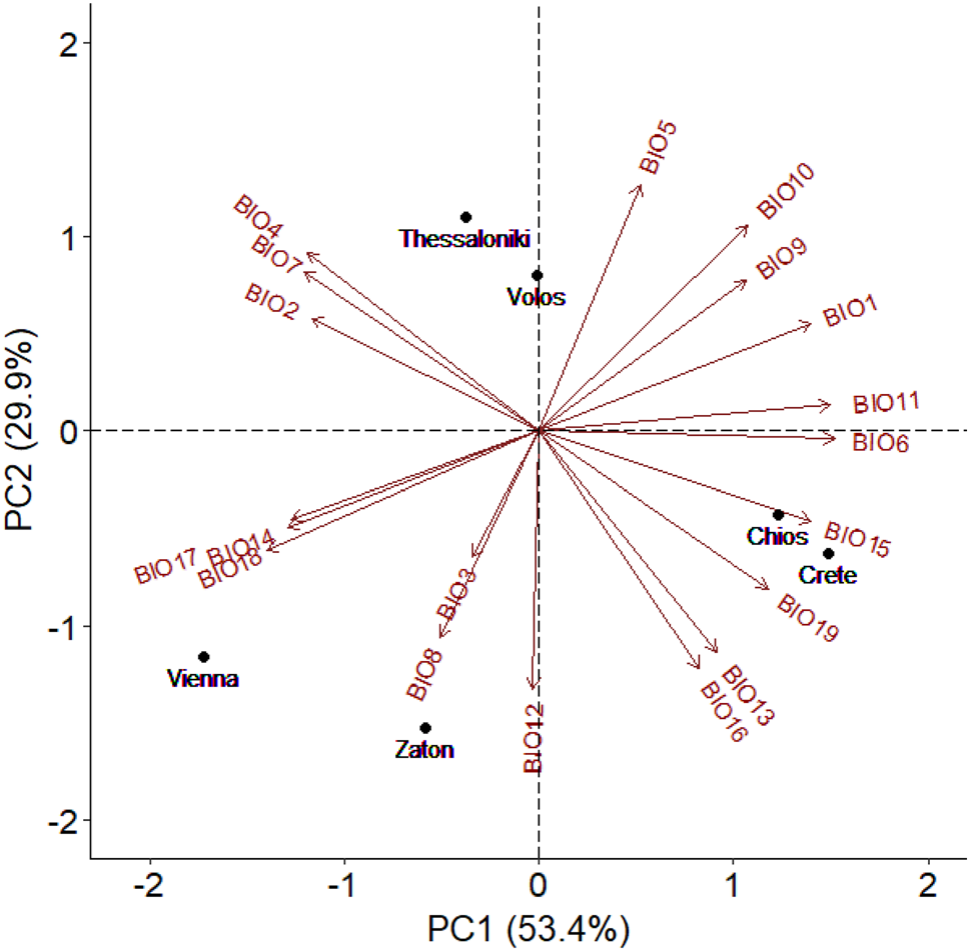
Climatic conditions in the six sampling sites. Principal component analysis (PCA) was performed on 19 variables describing environmental conditions in medfly collecting sites. The length and directionality of arrows (vectors) illustrate, respectively, the strength of contribution (loading) and sign of correlation (positive/negative) of each variable on the two components (PC1: x-axis, PC2: y-axis). Variables closer to each other are more correlated on a given component.

Bioclimatic variables of the first principal component (PC1) are mostly based on temperature and precipitation patterns separating colder from warmer sites (Fig. 2). Positive values of PC1, indicate regions with warmer temperatures and lower precipitation, while negative values of PC1 indicate regions with cooler temperatures and higher precipitation. Variables with the highest contribution on PC1 included annual mean temperature (BIO1), minimum temperature of coldest month (BIO6), mean temperature of coldest quarter (BIO11), low precipitation of driest month (BIO14), precipitation seasonality (coefficient of variation) (BIO15), precipitation of driest quarter (BIO17) and low precipitation of warmest quarter (BIO18) (see Supplementary Table S13).

PC2 provides insights into the seasonal distribution of precipitation in different regions (Fig. 2). A higher PC2 value suggests higher temperature variability within the region. Variables with the highest contribution on PC2 include maximum temperature of warmest month (BIO5), average temperature of wettest quarter (BIO8), and average temperature of warmest quarter (BIO10) as well as annual precipitation (BIO12) and precipitation of the wettest month (BIO13) and, precipitation of the wettest quarter (BIO16) (see Supplementary Table S13). The inclusion of both temperature and precipitation variables in the contributing factors to PC2 suggests that regions with high PC2 values, experience seasonal shifts in both temperature and precipitation patterns. This could imply distinct wet and dry seasons or fluctuations in temperature and precipitation between different quarters or months.

Regions with high PC1 and low PC2 values are characterized by warm temperatures and low precipitation, but with relatively constant temperature and precipitation throughout the year. On the other hand, regions with high PC1 and PC2 values have more variable temperature and precipitation patterns throughout the year.

Overall PCA analysis clusters the areas of origin of the tested populations to three groups (a) mainland central northern Greece (Thessaloniki and Volos), (b) eastern Mediterranean islands (Chios and Crete) and (c) northern areas (Vienna and Zaton) (Fig. 2).

#### Effect of bioclimatic variables on egg to adult survival rates

**Figure 3:**
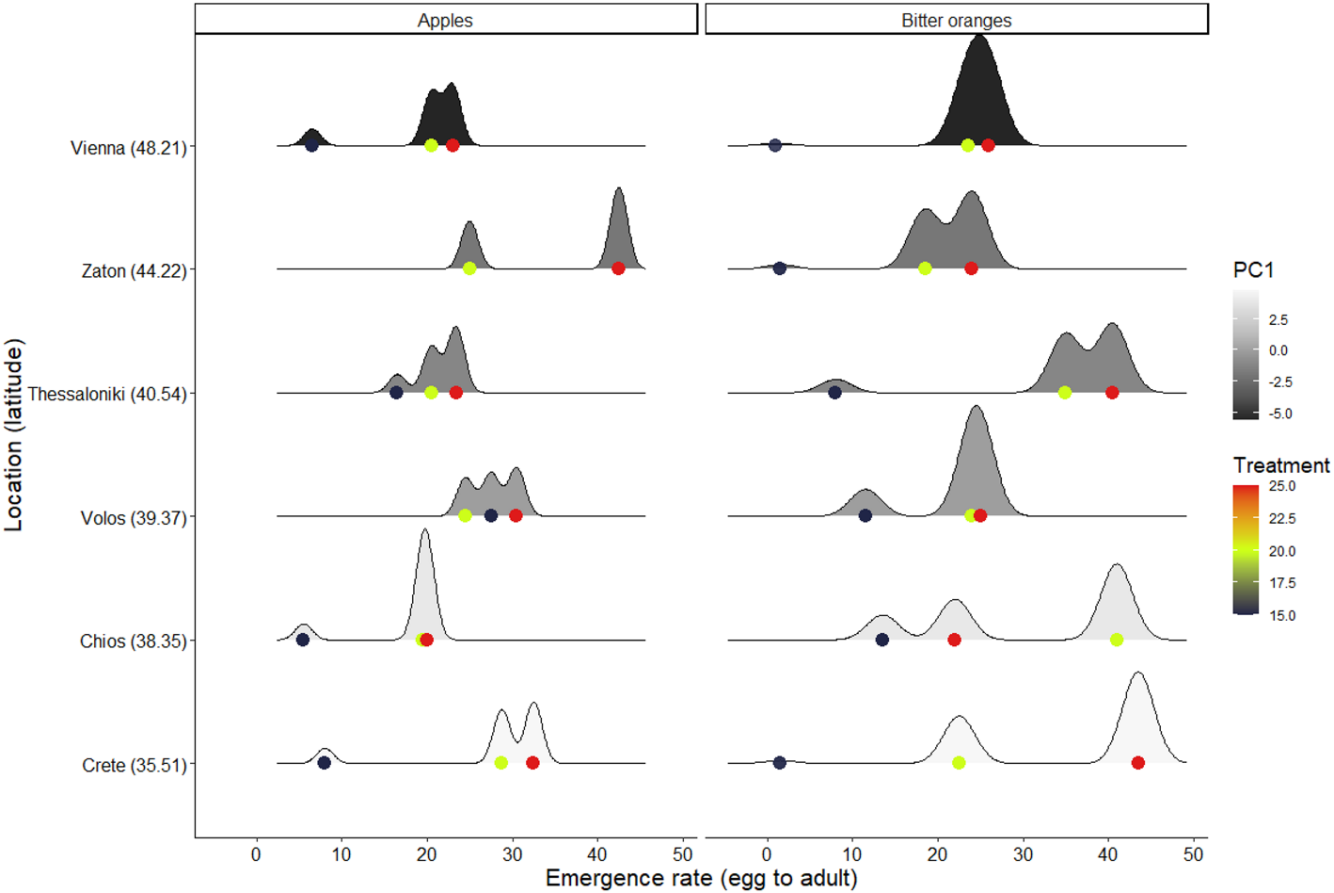
Density plots for the relationship between latitude and emergence rate of six different *C. capitata* populations. The effect of latitudinally varying temperatures and summer precipitation (PC1) is illustrated in grey scale (darker colors represent cooler locations with wet summer and the lighter ones the warmer locations with dry summers). The effect of temperature is illustrated in colored scale (red color represents 25 °C, green color represents 20 °C and dark blue represents 15 °C). The peaks of density plots show where values are concentrated.

The model, which distinguishes among latitude (as proxy of photoperiod and south to north invasion), and climatic variables, included PC1, PC2, host and temperature as well as their interaction as explanatory factors is given in Supplementary Table S14. The best fit model based on BIC revealed that macroclimatic conditions grouped in PC1, differentiating among the cold and warm sites, affected egg to adult survival rates (*P* < 0.001) (Fig. 3, Table 3). Similarly, thermal variability and winter precipitation of the population origin sites (PC2) (*P* = 0.048), was also related with egg to adult emergence rates. Latitude which can be considered as a proxy of the south to north invasion was also associated with survival rates (*P* < 0.01). The effect of latitude on survival rates varied across the different levels of PC1, and PC2 (Table 3). Egg to adult emergence rates were higher in warmer sites (positive PC1 value), with lower latitude compared to colder sites (negative PC1 values). Similarly, the effect of the host on survival rates varied significantly depending on the different values of PC1 (*P* < 0.001) and PC2 (P < 0.001) as indicated in Table 3. Finally, the effect of temperature on survival rates varied across PC1 and PC2 values (*P* < 0.001).

**Table 3:** Linear regression model results of the best model on the effects of latitude, bioclimatic variables (PC1, PC2) on egg to adult survival rates of *C. capitata.*

Variables in the model	Wald’s *x*^*2*^ (df)	B (95%CI)	*P*
Latitude	159.687(1)	15.488 (12.389, 18.587)	< 0.001
PC1	78.835 (1)	11.211 (7.627, 14.796)	< 0.001
PC2	3.920 (1)	− 12.326 (− 21.314, − 3.338)	0.048
Temperature	204.180 (1)	40.780 (35.122, 46.437)	< 0.001
Host	334.762(1)	− 360.044 (− 398.613, − 321.475)	< 0.001
Latitude * PC1	28.574(1)	0.780 (0.050, 0.107)	< 0.001
Latitude * PC2	26.440(1)	0.549 (0.340, 0.758)	< 0.001
Latitude * temperature	189.403(1)	− 0.939 (− 1.119, − 0.840)	< 0.001
Latitude * host	306.576(1)	8.337 (7.440, 9.315)	< 0.001
PC1 * temperature	175.550(1)	− 1.002 (− 1.150, − 0.854)	< 0.001
PC1 * host	338.593(1)	9.457 (8.449, 10.464)	< 0.001
PC2 * temperature	250.085(1)	− 0.680 (− 0.764, − 0.596)	< 0.001
PC2 * host	550.870(1)	6.482(5.941,7.023)	< 0.001
Temperature * host	165.778(1)	1.105 (34.071, 38.835)	< 0.001

Model selection was based on Bayesian Information Criterion (BIC) results (see Supplementary Table S14). “df”: degrees of freedom.

#### Effect bioclimatic variables on egg to adult developmental duration

**Figure 4:**
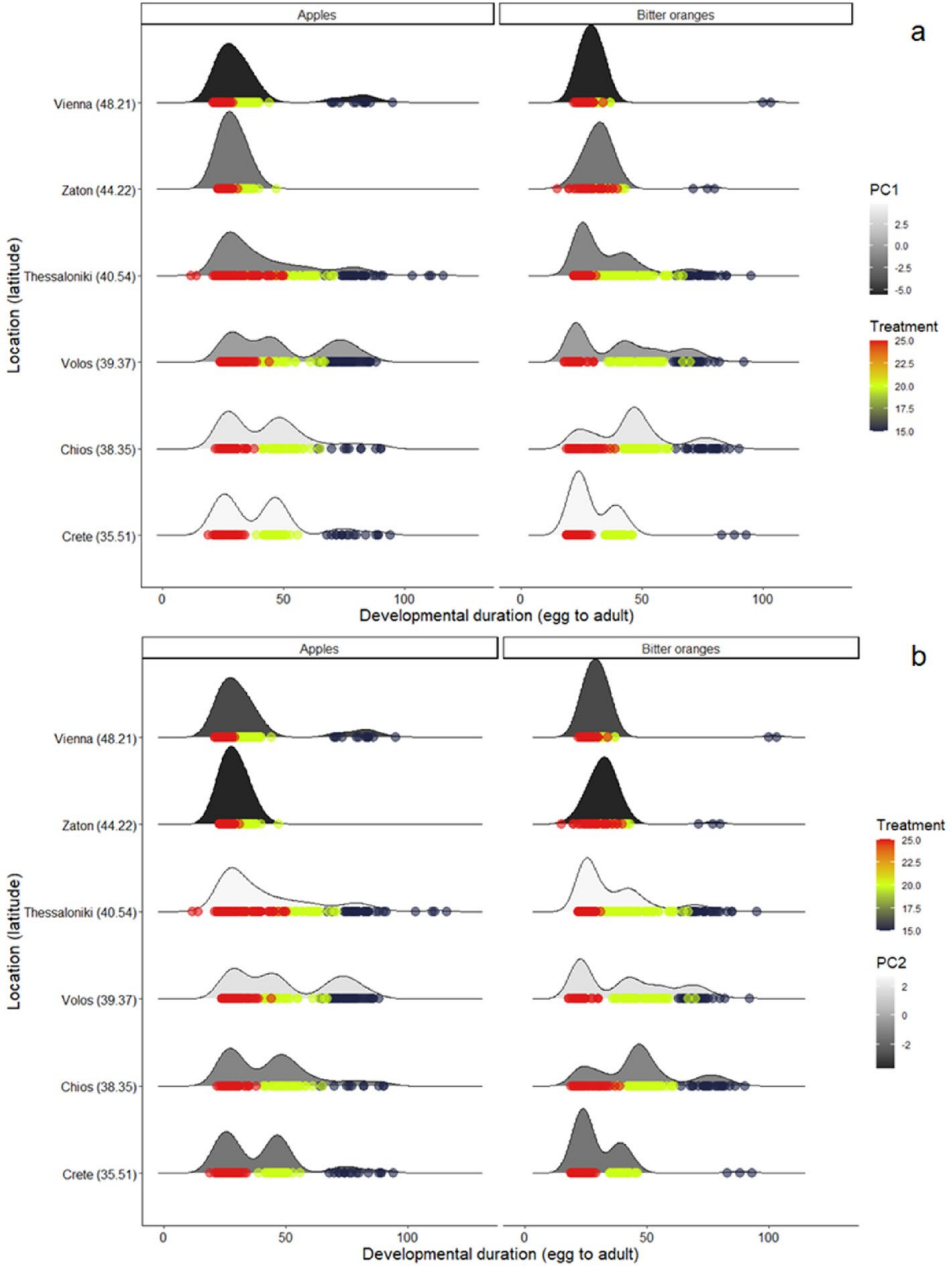
(**a**) Density plots for the relationship between latitude (as a proxy of photoperiod) and developmental duration of six different *C. capitata* populations. The effect of latitudinally varying temperatures and summer precipitation (PC1) is illustrated in grey scale (darker colors represents the colder locations with wet summer and the lighter ones the warmer locations with dry summers). The effect of temperature is illustrated in colored scale (red color represents 25 °C, green color represents 20 °C and dark blue represents 15 °C). The peaks of density plots show where values are concentrated. (**b**) Density plots for the relationship between latitude (as a proxy of photoperiod) and developmental duration of six different *C. capitata* populations. The effect of annual temperature range and seasonal precipitation of the year (PC2) on *C. capitata* populations are illustrated in grey scale (lighter colors represent locations, with higher temperature variation and lower winter precipitation and the darker ones the locations with lower temperature variation and higher winter precipitation). The effect of temperature is illustrated in colored scale (red color represents 25 °C, green color represents 20 °C and dark blue represents 15 °C). The peaks of density plots show where values are concentrated.

The model, distinguishing among latitude, and bioclimatic variables, included PC1, PC2, host and temperature as well as their interaction as explanatory factors is given in Supplementary Table S15. The best fit model based on BIC revealed that macroclimatic conditions differentiating among the cold and warm sites (PC1) (*P* < 0.001), is a significant predictor of the egg to adult developmental period (Fig. 4a, Table 4). Regions located at cold climates (Vienna, Zaton) (low PC1 values) and warm climates (Crete) (high PC1 values), were associated with shorter developmental duration compared to Thessaloniki, Volos and Chios where the developmental duration of immatures was longer and with higher variability (long queue in Fig. 4a). Additionally, thermal variability and winter precipitation among the collection sites of *C. capitata*, as indicated by PC2 (*P* < 0.001; Fig. 4b) affected the developmental duration of immatures. In regions with positive PC2 values higher temperature variation, is related with longer developmental durations compared to regions with negative PC2 values, where temperature variation was lower (Fig. 4b). Egg to adult developmental duration was significantly affected by the interaction between latitude with macroclimatic conditions (latitude * PC2, *P* = 0.018).

**Table 4:** Linear regression model results of the best model on the effects of latitude, bioclimatic variables (PC1, PC2) on egg to adult developmental duration of *C. capitata.*

Variables in the model	Wald’s *x*^*2*^ (df)	B (95%CI)	*p*
Latitude	19.788 (1)	7.775 (4.077, 11.473)	< 0.001
PC1	40.800 (1)	12.175 (8.227, 16.124)	< 0.001
PC2	16.713 (1)	22.267 (11.591, 32.942)	< 0.001
Temperature	7.102 (1)	9.102 (2.285, 15.920)	0.008
Host	31.496 (1)	− 57.922 (− 78.151, − 37.693)	< 0.001
Latitude * PC2	5.561 (1)	− 0.300 (− 0.549, − 0.051)	0.018
Latitude * temperature	15.025 (1)	− 0.333 (− 0.501, − 0.164)	< 0.001
Latitude * host	20.760 (1)	1.147 (0.654, 1.641)	< 0.001
PC1 * temperature	27.338 (1)	− 0.476 (− 0.655, − 0.298)	< 0.001
PC1 * host	14.515 (1)	1.102 (0.535, 1.669)	< 0.001
PC2 * temperature	52.039 (1)	− 0.373 (− 0.475, − 0.272)	< 0.001
Temperature * host	13.371 (1)	0.374 (0.174, 0.575)	< 0.001

Model selection was based on Bayesian Information Criterion (BIC) results (see Supplementary Table S15). *df* degrees of freedom.

## Discussion

Our findings suggest that the capacity to adapt to various overwintering hosts and temperature conditions is related to the geographic origin of medfly populations. This might be due to genetic variations among populations or differences in environmental conditions that populations have previously experienced. Linking these differences among the populations with the macroclimatic conditions, we found that both regional climatic variability and latitude can influence the performance of medfly populations. Regional climatic variability (e.g., temperature, humidity and rainfall), can affect the development and survival of *C. capitata* populations. On the other hand, latitude, that is directly related with the photoperiod, may be also related with a south to north invasion process of the pest. Hence, both invasion history that is shorter in northern areas as well as climate related selection pressures seem to interact and define the performance of medfly in different overwintering hosts and temperatures. Overall populations express different levels of plasticity in response to overwintering hosts and temperatures as far as developmental duration is regarded, which is decisive trait for overwintering survival in cooler areas^21^.

The variability in survival rates observed among populations could be attributed to a combination of factors, including genetic differences among populations, the invasion history, and other elements, such as the composition of gut bacteria communities^35–37^. Similar results have been reported by Diamantidis and colleagues^15^, under common garden experimental conditions considering medfly populations obtained from a much larger geographic scale. Mortality rates were in general higher than 60% for the different populations, which agrees with previous studies demonstrating high immature mortality of medflies in controlled laboratory conditions^20,26,27,38^. Mortality was higher in larvae compared to pupae. Active feeding of larvae exposes them to physical and chemical stress factors of the host fruit that increase death risk in contrast to pupal stage that is protected and “passive”. High larval mortality has been attributed to the toxic properties of the fruit rind such as the essential oils of citrus fruit^39^. However, in our study eggs were inserted in the flesh of both fruits and hence have not been exposed to toxic properties of the peel of fruit like bitter orange^39^.

While bitter oranges are among the favored hosts for *C. capitata*, survival rates of its immature stages were higher when reared in apples. Also, developmental duration was longer when immatures reared in apples. This comes in agreement with earlier studies^16,20,38^. The nutrient composition, the physicochemical characteristics, and the resistance to penetration are crucial factors that impact the vulnerability of the fruit and affect the performance of medfly immature stages^16,40–42^. Additionally to nutritional factors, host fruit cultivar and ripening stage can also affect the developmental duration^20,43^. In hosts such as figs, peaches and oranges, during the ripening process, protein levels increase, promoting shorter developmental duration^22^. Nash and Chapman^31^, demonstrated that decreases in both the quantity and the quality of protein had significant impact on the growth of medfly larvae, leading to higher mortality rates and longer developmental periods. Similarly changes in the quality of carbohydrates affected mortality during the pupal stage. Our findings align with the results of this study, confirming that *C. capitata* expresses developmental plasticity by adapting to various nutritional conditions. Larvae exhibit a strong capacity to adjust their growth rates by changing the expression of the endocrine system in response to varying diets^31^. This plasticity in dietary responses plays vital role in shaping trade-offs and interactions with environmental factors^44^.

Within the range of the tested temperatures (15–25 °C), the lower the temperature the lowest the survival rates of medfly immatures, indicating that *C. capitata* is well adapted to warmer climates. This comes in agreement with previous studies where low temperatures were prejudicial for egg to adult survival^45,46^. On the other hand, Dionysopoulou and colleagues^16^, found that lower temperatures, promoted survival rates. This difference in results is likely attributed to the fact that in this study first instar larvae were used to determine the development and survival of laboratory adapted populations, in two hosts under different temperatures. Previous studies have identified specific temperature thresholds for different stages of medfly development, including egg, larva, pupa and adult stages^17,26,47,48^. Furthermore, cold tolerance may vary among immatures of medfly as demonstrated by Behadili and colleagues^49^. The survival of immature stages under various temperature regimes is an important aspect of the invasion process^50^. The ability to rapidly alter survival through phenotypic plasticity may also adjust the survival capacity of *C. capitata* when introduced to a novel environment^51^. Recent studies suggest that medflies exhibit considerable levels of phenotypic plasticity in terms of thermal tolerance^28,51,52^.

Immature stages reared on fruits kept at 15 °C, expressed longer developmental duration. Similar effects of low temperatures on the developmental duration have been observed in other studies as well^16,26,45,46,53^. Temperature influences a wide range of physiological processes and the activities of enzymes, including the rate at which molecules diffuse and the extent to which membrane lipids become unsaturated^54,55^. Sgrò and colleagues^47^, demonstrated that environmental conditions experienced across the developmental stages can influence plastic responses of insects generating both beneficial and costly effects. Warm conditions accelerate developmental rates, leading to a new generation consisting of immatures that are poorly adapted to winter conditions. Hence, poorly adapted individuals may struggle to survive in harsh winter conditions^56^. On the other hand, extended developmental duration caused by low temperatures, may result in extreme phenotypes that are crucial for winter survival^21^. Furthermore, longer developmental duration in conjunction with low winter temperatures, may enable larvae not only to persist throughout the winter but also to bridge the gap of available hosts between the autumn and spring^21^.

The relationship between population growth, survival, developmental duration, and adaptation responses, is context dependent and influenced not only by the factors described above but also by the distinct microclimatic conditions *C. capitata* populations encounter in the wild. Understanding this relationship is essential for predicting how medflies respond to changing environmental conditions and their invasion success. In the present study, principal component analysis identified three distinct population subgroups (Vienna-Zaton, Thessaloniki-Volos, Chios-Crete), based on bioclimatic indicators. These associations likely stem from varying host availability, seasonal and diurnal responses to climate fluctuations and factors influencing overwintering strategies. In Europe, frost events are more frequent during spring, particularly in regions around the 40° latitude^57^. These frost events pose a risk to the most vulnerable stage of insects life cycle and can significantly impact insect population dynamics^24^. Nyamukondiwa and colleagues^51^, explored how thermal biology can affect population abundance of *C. capitata*, and demonstrated that low temperatures (LLT50:0 °C for 8h) can lead to collapses in adult populations of *C. capitata.* On the contrary, Manrakhan and colleagues^58^, found that medfly immature stages exhibit higher resistance to cold treatment, with 100% mortality observed after exposure to 0 °C for 9 days. These variations in cold tolerance among developmental stages of *C. capitata* have implications for the overwintering capacity of *C. capitata* populations. As the latitude increases and the temperature decreases, medflies use different overwintering strategies to cope with low temperatures, and the medfly’s overwintering capacity decreases due to several factors such as (a) reduced metabolic activity, (b) increased mortality, (c) lower availability of hosts^17,19,59^.

In the present study, the interaction between population origin and host indicated differential plasticity in developmental duration of immature stages among populations and probably adaptation to different hosts. Evidently, certain populations (like Thessaloniki, Volos and Crete) prolonged their developmental duration when reared in apples as opposed to bitter oranges, whereas others like Chios exhibited no differences in developmental duration between the two hosts. The lower temperature in combination with apples generated some extreme phenotypes that completed development in approximately four months (e.g., Thessaloniki). This aligns with the findings of Papadopoulos and colleagues^21^, who demonstrated that the developmental duration of *C. capitata* immatures in apples, under field conditions in Thessaloniki, spanned from October to the following March. Remarkably, our data revealed that not all populations could extend immatures developmental duration and apparently, they were not all able to overwinter as larvae in fruits. Plasticity in developmental duration was lower in populations obtained from the northern areas of Zaton and Austria (regions with high latitude), compared with those from Thessaloniki, Volos and Chios (regions with low latitude). Hence, it appeared that the Greek populations had evolved to survive winter conditions in larval stage, unlike those from more northern regions.

In Vienna, cold winters with low minimum temperatures along with frost events during the coldest season, create unfavorable conditions for the survival of medfly as indicated by Egartner and colleagues^60^. On the other hand, urbanization changes the natural environment and creates climatic conditions that can impact the insects physiology^61^. Buildings and structures in urban areas can create protected environments with more favorable climate conditions for insects. This phenomenon is often referred to as the "urban heat island" effect and can have both positive and negative impacts on overwintering of insects^62^. Heat islands can lead to changes in the thermal biology traits of insects, such as increased survival rates during the winter in regions where, under normal circumstances, these insects would not be able to withstand the low winter temperatures^63^. Rigamonti and colleagues^64^, demonstrated that in regions with low temperatures medfly can overwinter in “shelters” that provide protection from outdoor climatic conditions. Recent data demonstrate that medfly overwinters in protected areas in Vienna as adult and not as larvae within fruit (Ergartner and colleagues (2023), personal communication). Similar overwintering strategy has been found in the area of Lombardia (northern Italy)^65^. In Central Dalmatia, Bjeliš and colleagues^66^, demonstrated the successful overwintering of medflies as pupae in the wild (females emerging from overwintering pupae survive August next year) and as adults in urban areas. On the other hand, in regions from the Mediterranean Basin (Thessaloniki, Volos, Chios), *C. capitata* can overwinter mainly as larvae within fruits. In Thessaloniki, Papadopoulos and colleagues^21^, demonstrated that *C. capitata* overwinters almost exclusively in the larvae stage inside apples. Similarly, the presence of bitter oranges during the winter in Volos, enables medfly larvae to overwinter inside the fruits (Papadopoulos and colleagues, unpublished data). In Chios, medflies overwinter as 1st and 2nd instars within bitter oranges^67^. The lower plasticity in the Crete population may reflect adaptation to warmer conditions and overwintering in all developmental stages^18^. Since the origin of medfly populations varied not only in latitude but also in phytogeographic conditions (host type), this variation may have important consequences for the survival and developmental duration of *C. capitata* immature stages. Indeed, citrus are cultivated in Crete, Chios and Volos, whereas there are not present in Thessaloniki and Vienna. Finally, plasticity in pupae developmental duration is rather low for all tested populations except that of Crete, which may also indicate capacity to overwinter in this stage. Considering that continuous detection in Vienna have been reported the last two decades^60^ while the progress of invasion to northern Adriatic coast (Zaton) is more recent^66^, these findings suggest that in regions where *C. capitata* is not historically established, ability to respond and adapt to environmental changes is limited most probably due to reduced genetic diversity. On the other hand, in regions where medfly is already established, *C. capitata* has undergone a process of natural selection and adaption, which enables them to exhibit more effective responses to environmental changes^14,68^.

Overall, understanding how climate change and biological traits can affect *C. capitata* survival and development under different temperature conditions and in different hosts, is crucial for predicting the potential geographical range of this invasive species. The climate change, the international trade and the human movement may have favored the colonization and establishment of non-native species in environments where otherwise would not occur. Hence, the survival rates of medflies in temperate environments can be affected by even a little increase in average temperature since temperature affects the duration of cooling periods. Additionally, when the fruiting season is extended by global warming and tropical-subtropical fruits and vegetables are available in cooler areas, *C. capitata* is more likely to survive and thrive^69^. This is the first study addressing geographical patterns of survival and development of the widespread pest *C. capitata*. The results revealed that geographically isolated populations exhibited different survival rates in two overwintering hosts and different temperature regimes. Similarly developmental duration was prolonged in populations originating from lower latitudes. Plastic responses to development in the two overwintering hosts under different temperatures were population specific. Hence, our data provide novel insights into overwintering capacity of medfly and generate important data for predicting the expansion of this pest into cooler more temperate areas of Europe.

## Methods

### Insect populations and rearing conditions

Six *C. capitata* populations originating from Austria (Vienna), Croatia (Zaton) and Greece (Thessaloniki, Volos, Chios, Crete) were tested (Supplementary Fig. S4, Table 5). Population sampling sites spanning from ~ 35° to 48°N latitude with up to 157 m altitude to avoid altitudinal clines^24^. Pupae from Croatia and Austria were transported by a courier agent to our laboratory in Volos, Greece. To establish experimental colonies in the laboratory, approximately 1000 pupae from each location site were used. Populations originating from Greece, derived from field-infested oranges collected around Volos, Chios and Crete and from apples in case of Thessaloniki. Population originating from Croatia, derived from infested figs collected in Zaton (close to Zadar), and Austria, derived from infested pome fruits collected in Vienna. All experiments were conducted in the laboratory of Entomology and Agricultural Zoology at the University of Thessaly from September 2019 to January 2021.

**Table 5:** Latitude and longitude parameters of *C. capitata* collection sites.

Population	Latitude DMS	Longitude DMS
Vienna, Austria	48^o^12′29.43″N	16^ο^22′25.75″Ε
Zaton, Croatia	43^o^47′16.3″N	15^o^49′21.71″E
Thessaloniki, Greece	40^ο^38′24.23″Ν	22^ο^56′39.91″Ε
Volos, Greece	39^ο^21′43.88″Ν	22^ο^56′31.77″Ε
Chios, Greece	38^ο^22′15.53″Ν	26^o^8′10.85″E
Crete, Greece	35^o^30′49.79″N	24^o^1′4.93″E

Colonies of approximately 100 adults per cage from the populations used in the present study, were kept in wooden (30 × 30 × 30 cm), wire-mesh cages at 25 ± 1 °C, 45–55% relative humidity and 14:10 L:D photoperiod (photo phase started at 07:00 a.m.). Daylight fluorescent tubes with light intensity ranging from 1500 to 2000 Lux. Flies had free access to water and a standard adult diet (mixture of yeast hydrolysate, sugar and water at 1:4:5 ratio). Females were allowed to lay eggs on red, hollow, punctured plastic domes with diameter 5.5 cm and thickness 1 mm. Plastic domes were fitted in the lid of plastic Petri dish. In the base of petri dished, water was added to preserve the relative humidity. To stimulate oviposition, 0.5 ml of fresh orange juice, was added in a small cup and placed in the base of the petri dish^70^. Oviposition domes were placed in rearing cages for 24 h to collect the required number of eggs needed for the experiments. Eggs (50–100) were seeded in the artificial diet (50 g sugar, 50 g brewer’s yeast, 25 g soybean flour, 1g salt mixture, 4 g ascorbic acid, 4 g citric acid, 0.75 g sodium propionate and 250ml water). Flies from Zaton, Thessaloniki, Volos and Chios used, reared up to four generations (F1-F4), flies from Crete reared up to F8-F9 generations and flies from Vienna were reared up 3 generations from the time arrived at the laboratory.

### Fruit hosts

Pre adult survival and development of different medfly populations were assessed within two key overwintering hosts, apples, and bitter oranges. Apples (Golden Delicious cultivar) and bitter oranges (local cultivar) were collected from organic orchards of Naousa (Northern Greece) and Lechonia (near to Volos), respectively. All collected fruits were washed with tap water and stored at 6 ± 2 °C. Fruits have been remained under standard laboratory conditions (25 °C ± 1 °C, 45–55% relative humidity and 14:10L:D photoperiod) for at least 24h before being assigned to different temperatures. Collection of plant material, complied with relevant institutional, national, and international guidelines and legislation.

### Survival and development of immature stages

For each population, freshly laid eggs (up to 24 h) that were taken from the oviposition domes were implanted into the two host fruits (apples, bitter oranges). For this purpose, two artificial holes, approximately 1 mm in diameter, were drilled on opposite sides on the upper part of each host fruit, and 5 eggs were placed gently into each hole. Then, the infested fruits (10 eggs/fruit) were individually placed in plastic containers on a layer of sterilized sand and were covered with organdie cloth and transferred in the 3 different constant temperature conditions (15, 20, 25 °C). Daily, all artificially infested fruits were carefully inspected, and all newly formed pupae were recorded and collected. For each population, 20 replications (1 fruit with 10 eggs) per temperature /host (apples, bitter oranges) were used. Hence, 360 apples and 360 bitter oranges in total (20 for each of the 6 medfly populations and the three constant temperatures).

### Statistics

#### Effect of host, population sex and temperature on survival rates and developmental duration of *Ceratitis capitata*

Linear Regression models were used to determine the effect of host, population, sex and temperature on the emergence rate and developmental duration of egg to adult period. In both cases, the least significance difference (LSD) test was employed to adjust for multiple comparisons. The mean difference was considered significant at the 0.05 level. All data analyses and descriptive statistics were conducted using the statistical software SPSS 29.0 (IBM Corp, Armonk, NY, USA).

#### Effect of bioclimatic variables on survival rates and developmental duration of *Ceratitis capitata*

Statistical analysis was conducted using R version 4.1.1 (R Development Core Team 2022, R Foundation for Statistical Computing, Vienna, Austria)^71^. To characterize macroclimatic conditions of the six collection sites, we extracted 19 bioclimatic variables (Bioclim1-19) related to temperature and precipitation from the WorldClim database (Version 2.1, http://www.worldclim.org) using latitudinal and longitudinal coordinates for each site (0.5 min spatial resolutions; current data 1970–2000) (see Supplementary Table S10)^72^^.^ Principal component analysis (PCA) on 19 bioclimatic variables was performed to investigate environmental variation across fly populations. Multivariable linear regression models were used to assess the effect of latitude, host, temperature, PC1 and PC2 on pupation rates and developmental durations. For the model building procedure, initially, an exhaustive search was employed to find the best model regarding adjusted R^2^, Akaike information criterion (AIC) and Bayesian information criterion (BIC) among all possible models without any interaction terms using the function “regsubsets” (package: leaps)^73^. Then, all possible models with two-way interactions were examined using the function “ols step all possible” (package olsrr)^74^, and the best biological meaningful models were selected among those with the highest adjusted R^2^, and the lowest AIC and BIC for all responses variables.

### Supplementary Information

Supplementary Information.

## Data Availability

The datasets used and/or analyzed during the current study will be available from the corresponding author on reasonable request.
