# Supplementary material for: Latitudinal variation in survival and immature development of Ceratitis capitata populations reared in two key overwintering hosts
Source: Sci Rep. 2024 Jan 3;14:467. doi: 10.1038/s41598-023-50587-2 (PMC10764328; doi:10.1038/s41598-023-50587-2)
Supplement: Supplementary file 1 — Supplementary Information. [file 41598_2023_50587_MOESM1_ESM.docx]

**Supplementary Information**

**Supplementary Table S1:** Pairwise comparisons (host *population) of estimated marginal means based on egg to adult survival rate of *C. capitata* populations. The mean difference is significant at the level 0.05.

| **Comparison groups** | **Mean Difference ± SE** | **df** | | ***P*** |
| --- | --- | --- | --- | --- |
| Apples_Vienna*B. oranges_Vienna | -3.247 ± 0.681 | | 1 | <0.001 |
| Apples_Zaton*B.oranges_Zaton | 9.827 ± 0.661 | | 1 | <0.001 |
| Apples_Thes/niki*B.oranges_Thes/niki | -13.402 ± 0.409 | | 1 | <0.001 |
| Apples_Volos*B.oranges_Volos | 5.514 ± 0.580 | | 1 | <0.001 |
| Apples_Chios*B.oranges_Chios | -13.695± 0.644 | | 1 | <0.001 |
| Apples_Crete*B.oranges_Crete | -3.091 ± 0.599 | | 1 | <0.001 |

**Supplementary Table S2:** Effects of explanatory variables of the linear models on egg to adult emergence rates of *C. capitata* immature stages. Immatures from Crete reared in bitter oranges form the baseline.

| **Variables in the model** | **B ± SE** | **Wald x^2^** | **df** | ***P*** |
| --- | --- | --- | --- | --- |
| Intercept | -40.198 ± 2.606 | 237.914 | 1 | <0.001 |
| Host | 13.951 ± 1.692 | 67.944 | 1 | <0.001 |
| Population |  |  |  | <0.001 |
| Vienna | 26.820 ± 3.403 | 62.117 | 1 | <0.001 |
| Zaton | --6.306 ± 3.713 | 2.884 | 1 | 0.089 |
| Thes/niki | 31.243 ± 2.644 | 139.531 | 1 | <0.001 |
| Volos | 38.046 ± 2.748 | 191.562 | 1 | <0.001 |
| Chios | 53.335 ± 3.016 | 312.715 | 1 | <0.001 |
| Females | 1.241 ± 1.658 | 0.561 | 1 | 0.454 |
| Temperature | 3.274 ± 0.1108 | 873.030 | 1 | <0.001 |
| Host * Population |  |  |  | <0.001 |
| Apples * Vienna | -0.156 ± 0.906 | 0.030 | 1 | 0.863 |
| Apples * Zaton | 17.453 ± 0.892 | 382.739 | 1 | <0.001 |
| Apples * Thes/niki | -10.311 ± 0.725 | 202.174 | 1 | <0.001 |
| Apples * Volos | 8.606 ± 0.8392 | 105.164 | 1 | <0.001 |
| Apples * Chios | -10.604 ± 0.882 | 144.376 | 1 | <0.001 |
| Apples * Females | -0.365 ± 0.461 |  | 1 | 0.428 |
| Apples * Temperature | -0.774 ± 0.694 | 124.210 | 1 | <0.001 |
| Population * Sex |  |  |  | 0.998 |
| Population * Temperature |  |  |  | <0.001 |
| Vienna * Temperature | -1.601± 1.449 | 122.130 | 1 | <0.001 |
| Zaton * Temperature | -0.287± 0.157 | 3.305 | 1 | 0.069 |
| Thes/niki *Temperature | -1.274 ± 0.113 | 127.415 | 1 | <0.001 |
| Volos * Temperature | -2.123 ± 0.119 | 319.172 | 1 | <0.001 |
| Chios * Temperature | -2.423 ± 0.133 | 332.152 | 1 | <0.001 |
| Sex * Temperature |  |  |  | 0.524 |

**Supplementary Table S3:** Pairwise comparisons (host * population) of estimated marginal means based on egg to adult developmental duration of *C. capitata* populations. The mean difference is significant at the level 0.05.

| **Comparison groups** | **Mean Difference ± SE** | **df** | | ***P*** |
| --- | --- | --- | --- | --- |
| Apples_Vienna*B. oranges_Vienna | 2.40 ± 1.005 | | 1 | 0.017 |
| Apples_Zaton*B.oranges_Zaton | -2.94 ± 0.983 | | 1 | 0.003 |
| Apples_Thes/niki*B.oranges_Thes/niki | 5.00 ± 0.604 | | 1 | <0.001 |
| Apples_Volos*B.oranges_Volos | 3.32 ± 0.856 | | 1 | <0.001 |
| Apples_Chios*B.oranges_Chios | 1.71± 0.950 | | 1 | 0.071 |
| Apples_Crete*B.oranges_Crete | 4.43 ± 0.884 | | 1 | <0.001 |

**Supplementary Table S4:** Effects of explanatory variables of the linear models on egg to adult developmental duration of *C. capitata* immature stages. Immatures from Crete reared in bitter oranges form the baseline.

| **Variables in the model** | **B ± SE** | **Wald x^2^** | **df** | ***P*** |
| --- | --- | --- | --- | --- |
| Intercept | 131.622 ± 3.843 | 1173.277 | 1 | <0.001 |
| Host | 11.494 ± 2.495 | 21.217 | 1 | <0.001 |
| Population |  |  |  | <0.001 |
| Vienna | -21.032 ± 5.017 | 17.571 | 1 | <0.001 |
| Zaton | -58.982 ± 5.476 | 116.029 | 1 | <0.001 |
| Thes/niki | 0.788 ± 3.899 | 0.041 | 1 | 0.840 |
| Volos | 4.438 ± 4.0531 | 1.199 | 1 | 0.274 |
| Chios | 14.872 ± 4.447 | 11.184 | 1 | <0.001 |
| Sex | -0.741 ± 2.445 | 0.092 | 1 | 0.762 |
| Temperature | -4.393 ± 0.1634 | 723.323 | 1 | <0.001 |
| Host * Population |  |  |  | <0.001 |
| Apples * Vienna | -2.035 ± 1.336 | 723.323 | 1 | 0.128 |
| Apples * Zaton | -7.370 ± 1.315 | 2.321 | 1 | <0.001 |
| Apples * Thes/niki | 0.566 ± 1.069 | 31.390 | 1 | 0.596 |
| Apples * Volos | -1.118 ± 1.237 | 0.816 | 1 | 0.366 |
| Apples * Chios | -2.719 ± 1.301 | 4.367 | 1. | 0.037 |
| Host * Sex |  |  |  | 0.466 |
| Apples * Temperature | -0.313 ± 0.102 | 0.731 | 1 | 0.002 |
| Population * Sex |  |  |  | 0.026 |
| Vienna * Female | -0.332 ± 1.317 | 0.063 | 1 | 0.801 |
| Zaton * Female | 0.116 ± 1.289 | 0.008 | 1 | 0.929 |
| Thes/niki * Female | -0.698 ± 1.048 | 0.444 | 1 | 0.505 |
| Volos * Female | -2.277 ± 1.220 | 3.482 | 1 | 0.062 |
| Chios * Female | -3.508 ± 1.261 | 7.728 | 1 | 0.005 |
| Population* Temperature |  |  |  | <0.001 |
| Vienna * Temperature | 0.820 ± 0.214 | 14.754 | 1 | <0.001 |
| Zaton * Temperature | 2.634 ± 0.233 | 128.218 | 1 | <0.001 |
| Thes/niki *Temperature | 0.043 ± 0.166 | 0.067 | 1 | 0.796 |
| Volos * Temperature | -0.015 ± 0.175 | 0.008 | 1 | 0.930 |
| Chios * Temperature | -0.386 ± 0.100 | 3.887 | 1 | 0.049 |
| Sex * Temperature |  |  |  | 0.558 |

**Supplementary Table S5:** Pairwise comparisons (population * sex) of estimated marginal means based on egg to adult developmental duration of *C. capitata* populations. The mean difference is significant at the level 0.05.

| **Comparison groups** | **Mean Difference ± SE** | **df** | | ***P*** |
| --- | --- | --- | --- | --- |
| Vienna_Females*Vienna_Males | -0.04 ± 0.999 | | 1 | 0.967 |
| Zaton_Females*Zaton_Males | 0.41± 0.967 | | 1 | 0.675 |
| Thess/niki_Females*Thes/niki_Males | -0.41± 0.597 | | 1 | 0.495 |
| Volos_Females*Volos_Males | -1.99± 0.855 | | 1 | 0.020 |
| Chios_Females*Chios_Males | -3.22± 0.916 | | 1 | <0.001 |
| Crete_Females*Crete_Males | 0.29 ± 0.861 | | 1 | 0.736 |

**
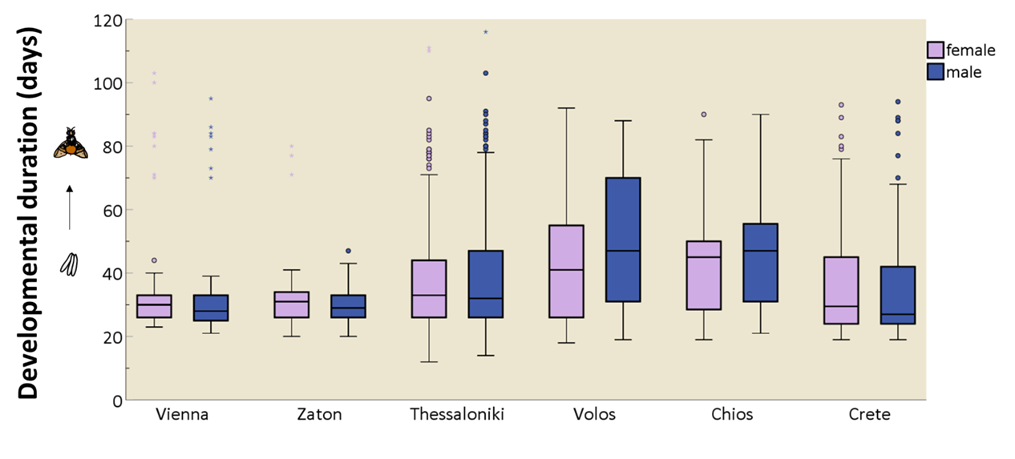
Supplementary Figure S1:** Effect of sex on developmental duration of egg to adult of different *C. capitata* populations. Boxplots include the median, the 1st and the 3rd quartile. Whiskers indicate the lowest-highest value inside the interval defined by ± the 1.5-fold interquartile range from the 1st/3rd quartile.

**Appendix 1.** Effect of temperature on survival rates of egg to pupa and pupa to adult of different *C. capitata* populations, in two different overwintering hosts (apples and bitter oranges).

**Survival rates**

*Egg to pupa*

Egg to pupa survival rates of all tested populations, in both host fruits held under the three constant temperatures (15,20,25^o^C) are given in Supplementary Figure 2a. Results indicated that all factors had a significant impact on the survival rates from egg to pupa (Supplementary Table S6). Survival rates varied among populations, and this variation differed depending on the host fruit (*P*< 0.001).Pairwise comparisons revealed that in apples egg to pupa survival rates were higher for pupae obtained from Zaton and Volos compared to bitter oranges (*P* < 0.001), while pupae obtained from Thessaloniki, Chios and Crete exhibited higher survival rates when reared in bitter oranges (*P* < 0.001).For pupae obtained from Vienna, survival rates were similar between the two hosts (*P* = 0.762) (Supplementary Table S7). Egg to pupa survival rates were also influenced by the interaction between host and temperature (*P* < 0.001). In apples an increase of temperature led to decreased survival compared to bitter oranges. Generally, at low temperatures (15^o^C), egg to pupae survival rates were higher in apples, whereas with the increase of temperature survival rates tend to be higher in bitter oranges. Finally, the interaction between population and temperature also had a significant effect on egg to pupa survival rates (*P* < 0.001). Across all tested populations, an increase in temperature generally led to higher survival rates, except for immatures originated from Chios, where survival rates were higher at 20^o^C compared to 25^o^C.

*Pupa to adult*

Pupa to adult survival rates of all tested populations, in both host fruits held under the three constant temperatures (15,20,25^o^C) are given in Supplementary Figure 2b. Results indicating the effects of tested factors on pupa to adult survival rates are given in Supplementary Table S6. Similarly with egg to pupa survival rates, results indicated that survival rates varied among populations, and this variation differed depending on the host fruit host fruit (*P*< 0.001). Pairwise comparisons revealed that pupa to adult survival rates were higher in apples compared to bitter oranges for flies obtained from Zaton, Volos and Crete (*P* < 0.001), while for adults obtained from Vienna, Thessaloniki and Chios, survival rates were higher when reared in bitter oranges (Supplementary Table S7). Further, the interaction host by temperature, significantly affected egg to adult survival rates (*P*< 0.001). Similarly with egg to pupa survival rates, in apples an increase of temperature led to decreased pupae to adult survival rates compared to bitter oranges. Generally, at low temperatures (15^o^C), survival rates were higher in apples, whereas with the increase of temperature survival rates tend to be higher in bitter oranges. Pupa to adult survival rates were differentially affected by the increase of temperature (*P* < 0.001) (Supplementary Table S6). Finally, the increase of temperature led to higher survival rates in females compared to males (*P* = 0.011).


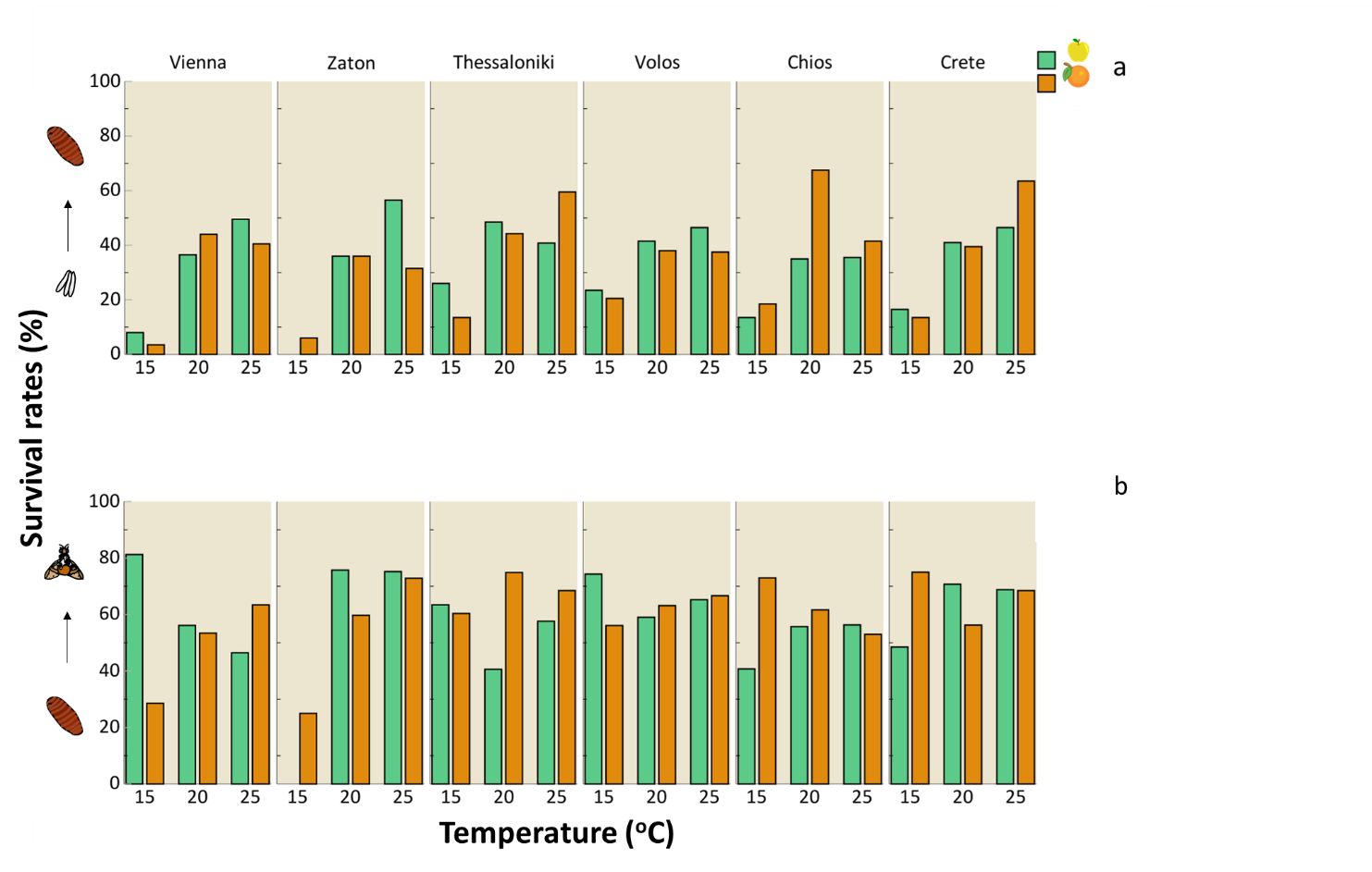


**Supplementary Figure S2:** Effect of temperature on survival rates of egg to pupae (a) and pupae to adult (b), of different *C. capitata* populations, in two different overwintering hosts (apples and bitter oranges). The length of each bar corresponds to the survival rates of each population, reared on the two hosts under different constant temperatures.

**Supplementary Table S6:** Results of linear models testing the effects of host, population, sex and temperature, on egg to pupa and pupa to adult survival rates.

| **Variables in the model** | **Wald *x*^2^** | **df** | ***P*** |
| --- | --- | --- | --- |
| *Egg to pupa* |  |  |  |
| Intrercept | 71.294 | 1 | <0.001 |
| Host | 44.557 | 1 | <0.001 |
| Population | 266.824 | 5 | <0.001 |
| Temperature | 2195.578 | 1 | <0.001 |
| Host * Population | 963.111 | 5 | <0.001 |
| Host * Temperature | 64.421 | 1 | <0.001 |
| Population * Temperature | 289.534 | 5 | <0.001 |
| *Pupa to adult* |  |  |  |
| Intercept | 2945.616 | 1 | <0.001 |
| Host | 48.338 | 1 | <0.001 |
| Population | 246.700 | 5 | <0.001 |
| Sex | 6.729 | 1 | <0.009 |
| Temperature | 18.836 | 1 | <0.001 |
| Host *Population | 1249.749 | 5 | <0.001 |
| Host * Sex | 0.176 | 1 | 0.675 |
| Host * Temperature | 64.812 | 1 | <0.001 |
| Population * Sex | 3.276 | 5 | 0.657 |
| Population * Temperature | 362.716 | 5 | <0.001 |
| Sex * Temperature | 6.445 | 1 | 0.011 |

**Supplementary Table S7:** Pairwise comparisons (host *population) of estimated marginal means based on egg to pupa and pupa to adult survival rates of *C. capitata* populations. The mean difference is significant at the level 0.05.

| **Comparison groups** | **Mean Difference ± SE** | **df** | | ***P*** |
| --- | --- | --- | --- | --- |
| *Egg to pupa* |  | |  |  |
| Apples_Vienna*B. oranges_Vienna | 0.262 ± 0.866 | | 1 | 0.762 |
| Apples_Zaton*B.oranges_Zaton | 14.384 ± 0.940 | | 1 | <0.001 |
| Apples_Thes/niki*B.oranges_Thes/niki | -5.290 ± 0.539 | | 1 | <0.001 |
| Apples_Volos*B.oranges_Volos | 4.258 ± 0.797 | | 1 | <0.001 |
| Apples_Chios*B.oranges_Chios | -20.992± 0.822 | | 1 | <0.001 |
| Apples_Crete*B.oranges_Crete | -7.225 ± 0.791 | | 1 | <0.001 |
| *Pupa to adult* |  | |  |  |
| Apples_Vienna*B. oranges_Vienna | -3.688 ± 0.845 | | 1 | <0.001 |
| Apples_Zaton*B.oranges_Zaton | 9.648 ± 0.813 | | 1 | <0.001 |
| Apples_Thes/niki*B.oranges_Thes/niki | -17.762 ± 0.509 | | 1 | <0.001 |
| Apples_Volos*B.oranges_Volos | 2.231 ± 0.720 | | 1 | <0.001 |
| Apples_Chios*B.oranges_Chios | -6.515± 0.799 | | 1 | <0.001 |
| Apples_Crete*B.oranges_Crete | 5.190 ± 0.742 | | 1 | <0.001 |

**Appendix 2.** Effect of temperature on developmental duration of egg to pupa (a) and pupa to adult (b), of different *C. capitata* populations, in two different overwintering hosts (apples and bitter oranges).

*Egg to pupa*

The developmental duration from egg to pupa of the six *C. capitata* populations, in both apples and bitter oranges, held under constant temperatures (15, 20, 25^o^C) is given in Supplementary Figure 3a. Results indicating the effect of the tested factors on egg to pupa developmental duration are given in Supplementary Table S8. Similarly, with survival rates, developmental duration among populations varied differently between the two hosts. Pairwise comparisons revealed that in all tested populations developmental duration was longer in apples with exception of Zaton, where developmental duration was longer in bitter oranges (*P* = 0.003) (Supplementary Table S9). Finally, the interaction between temperature and population, also had a significant effect on egg to pupa developmental duration (*P* < 0.001). Overall, in all tested populations the increase of temperature prolonged the developmental duration.

*Pupa to adult*

Dara presented in Supplementary Figure 3b (Supplementary Table S8), indicate the effect of host and temperature on the developmental duration from pupa to adult of the six different *C. capitata* populations tested. Among populations, developmental duration varied differently between apples and bitter oranges. In apples developmental duration from pupae to adult was longer in apples for Thessaloniki, Chios and Crete (*P* < 0.001, respectively), while in Vienna and Zaton, pupa to adult developmental duration was longer in bitter oranges. In Volos, pupa to adult developmental duration was similar between the two hosts (*P* = 0.114) (Supplementary Table S9). Finally, the significant interaction between population and temperature indicated that temperature affected in a different way developmental duration of different populations (*P* < 0.001). Overall, similarly with egg to pupa developmental duration, in all tested populations the increase of temperature prolonged the developmental duration. Finally, sex did not affect the developmental duration from pupa to adult of the six *C. capitata* populations, regardless of the tested temperature and host reared in (Supplementary Table 8).


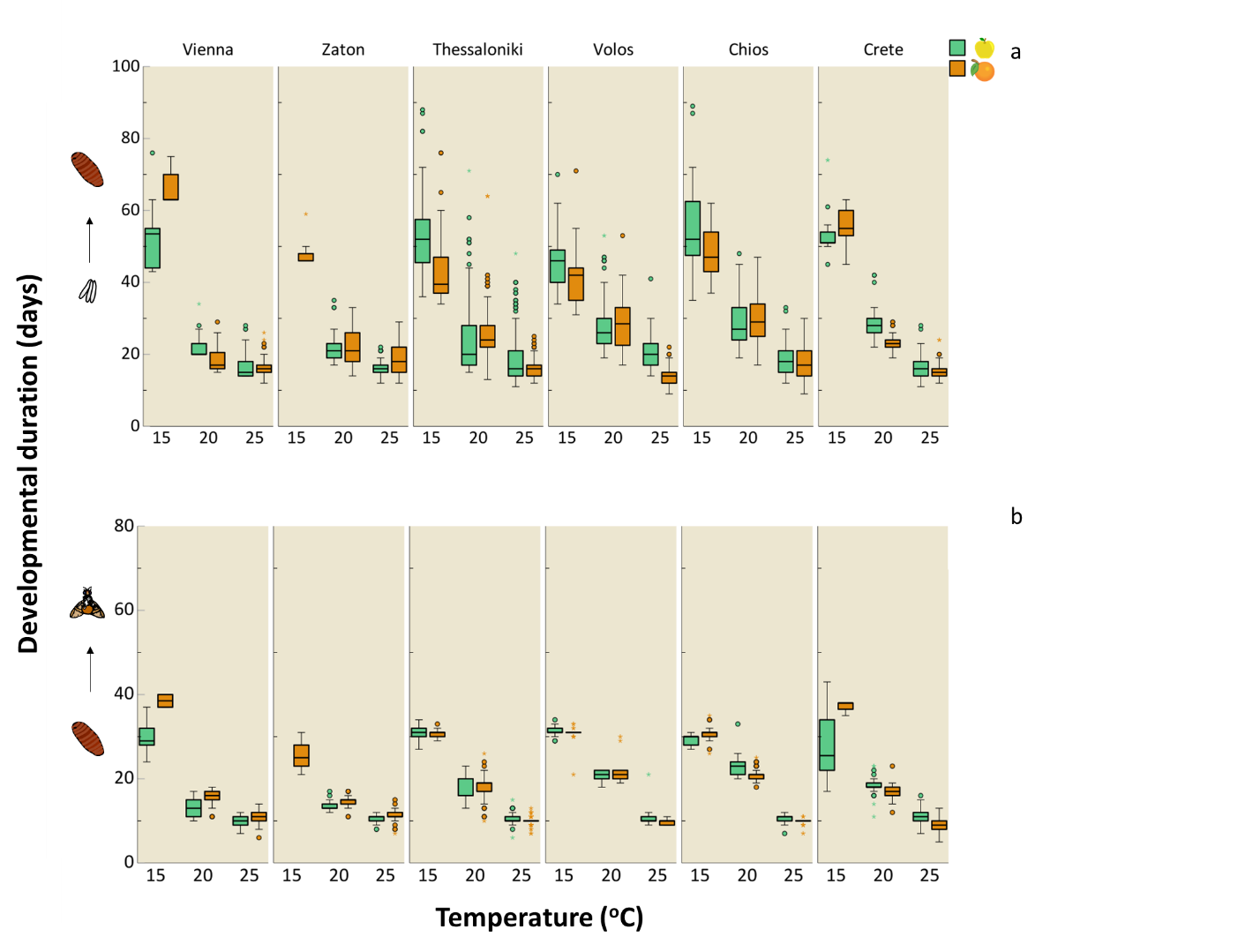


**Supplementary Figure S3:** Effect of temperature on developmental duration of egg to pupa (a) and pupa to adult (b) of different *C. capitata* populations, in two different overwintering hosts (apples and bitter oranges) under the three constant temperatures 15, 20 and 25^o^C. Boxplots include the median, the 1st and the 3rd quartile. Whiskers indicate the lowest-highest value inside the interval defined by ± the 1.5-fold interquartile range from the 1st/3rd quartile.

**Supplementary Table S8.** Results of linear models testing the effects of host, population, temperature, and sex on developmental duration of *C. capitata* immature stages.

| **Variables in the model** | **Wald *x*^2^** | **df** | **P** |
| --- | --- | --- | --- |
| *Egg to pupa* |  |  |  |
| Intercept | 7012.817 | 1 | <0.001 |
| Host | 4.655 | 1 | 0.017 |
| Population | 162.580 | 5 | <0.001 |
| Temperature | 3545.437 | 1 | <0.001 |
| Host*Population | 33.871 | 5 | <0.001 |
| Host*Temperature | 1.627 | 1 | 0.202 |
| Population*Temperature | 133.726 | 5 | <0.001 |
| *Pupa to adult* |  |  |  |
| Intercept | 18221.505 | 1 | <0.001 |
| Host | 1.192 | 1 | 0.275 |
| Population | 691.931 | 5 | <0.001 |
| Sex | 0.007 | 1 | 0.933 |
| Temperature | 9377.350 | 1 | <0.001 |
| Host*Population | 64.010 | 5 | <0.001 |
| Host*Sex | 0.242 | 1 | 0.623 |
| Host*Temperature | 1.632 | 1 | 0.201 |
| Population*Sex | 0.646 | 5 | 0.986 |
| Population*Temperature | 556.146 | 5 | <0.001 |
| Sex*Temperature | 0.010 | 1 | 0.920 |

**Supplementary Table S9:** Pairwise comparisons (host *population) of estimated marginal means based on egg to pupa and pupa to adult developmental duration of *C. capitata* populations. The mean difference is significant at the level 0.05.

| **Comparison groups** | **Mean Difference ± SE** | **df** | | ***P*** |
| --- | --- | --- | --- | --- |
| *Egg to pupa* |  | |  |  |
| Apples_Vienna*B. oranges_Vienna | 2.49± 0.756 | | 1 | 0.001 |
| Apples_Zaton*B.oranges_Zaton | -2.41 ± 0.820 | | 1 | 0.003 |
| Apples_Thes/niki*B.oranges_Thes/niki | 2.08 ± 0.471 | | 1 | <0.001 |
| Apples_Volos*B.oranges_Volos | 3.47 ± 0.696 | | 1 | <0.001 |
| Apples_Chios*B.oranges_Chios | 1.48 ± 0.717 | | 1 | 0.039 |
| Apples_Crete*B.oranges_Crete | 2.25 ± 0.690 | | 1 | 0.001 |
| *Pupa to adult* |  | |  |  |
| Apples_Vienna*B. oranges_Vienna | -1.00 ± 0.305 | | 1 | 0.001 |
| Apples_Zaton*B.oranges_Zaton | -1.29 ± 0.294 | | 1 | <0.001 |
| Apples_Thes/niki*B.oranges_Thes/niki | 0.63 ± 0.184 | | 1 | <0.001 |
| Apples_Volos*B.oranges_Volos | 0.41 ± 0.260 | | 1 | 0.114 |
| Apples_Chios*B.oranges_Chios | 0.75 ± 0.289 | | 1 | <0.001 |
| Apples_Crete*B.oranges_Crete | 1.17 ± 0.268 | | 1 | <0.001 |

**Supplementary Table S10:** List of the bioclimatic variables used in the PCA (WorldClim database v2.1,0.5 min spatial resolutions, current data 1970-20001; [www.worldclim.org](http://www.worldclim.org)).

| Variable | Description |
| --- | --- |
| BIO1 | Annual Mean Temperature |
| BIO2 | Mean Diurnal Range (Mean of monthly (max temp - min temp)) |
| BIO3 | Isothermality (bio2/bio7) (×100) |
| BIO4 | Temperature Seasonality (standard deviation ×100) |
| BIO5 | Max Temperature of Warmest Month |
| BIO6 | Min Temperature of Coldest Month |
| BIO7 | Temperature Annual Range (bio5-bio6) |
| BIO8 | Mean Temperature of Wettest Quarter |
| BIO9 | Mean Temperature of Driest Quarter |
| BIO10 | Mean Temperature of Warmest Quarter |
| BIO11 | Mean Temperature of Coldest Quarter |
| BIO12 | Annual Precipitation |
| BIO13 | Precipitation of Wettest Month |
| BIO14 | Precipitation of Driest Month |
| BIO15 | Precipitation Seasonality (Coefficient of Variation) |
| BIO16 | Precipitation of Wettest Quarter |
| BIO17 | Precipitation of Driest Quarter |
| BIO18 | Precipitation of Warmest Quarter |
| BIO19 | Precipitation of Coldest Quarter |

**Supplementary Table S11:** 19 bioclimatic variables for each site were extracted from WorldClim database v2.1 using latitudinal and longitudinal coordinates (0.5 min spatial resolutions; current data 1970-2000[^1^](#_ENREF_1); [www.worldclim.org](http://www.worldclim.org) ).

| Population | Long. | Lat. | BIO1 | BIO2 | BIO3 | BIO4 | BIO5 | BIO6 | BIO7 | BIO8 | BIO9 | BIO  10 | BIO  11 | BIO  12 | BIO13 | BIO14 | BIO15 | BIO16 | BIO17 | BIO  18 | BIO19 |
| --- | --- | --- | --- | --- | --- | --- | --- | --- | --- | --- | --- | --- | --- | --- | --- | --- | --- | --- | --- | --- | --- |
| Vienna | 16.3 | 48 | 9.9 | 9.6 | 0.32 | 72.46 | 26.1 | -3.7 | 29.8 | 19.2 | 1.8 | 19.2 | 0.5 | 623 | 75 | 35 | 28 | 213 | 115 | 213 | 118 |
| Zaton | 15.1 | 44 | 14 | 10 | 0.36 | 61.41 | 29.2 | 1.5 | 27.7 | 10.7 | 22 | 22 | 6.3 | 995 | 133 | 47 | 32 | 371 | 164 | 164 | 277 |
| Thes/niki | 22.9 | 39 | 16.1 | 9.8 | 0.31 | 74.31 | 32.6 | 1.6 | 31 | 8.4 | 25.2 | 25.7 | 6.6 | 440 | 53 | 20 | 27 | 144 | 72 | 76 | 128 |
| Volos | 22.9 | 39 | 16.9 | 10.6 | 0.34 | 69.28 | 33.7 | 3.3 | 30.4 | 10 | 26 | 26 | 8.3 | 491 | 71 | 12 | 43 | 186 | 54 | 54 | 176 |
| Chios | 26.1 | 38 | 17.9 | 8 | 0.32 | 59.57 | 32 | 7.1 | 24.9 | 10.7 | 25.7 | 25.7 | 10.7 | 624 | 145 | 1 | 89 | 367 | 14 | 14 | 367 |
| Chania | 24 | 35 | 19 | 6.2 | 0.31 | 51.93 | 29.5 | 9.5 | 20 | 12.7 | 25.7 | 25.7 | 12.7 | 619 | 122 | 1 | 84 | 329 | 6 | 6 | 288 |

**Supplementary Table S12:** Principal Components (PCs) with their Eigenvalues, variance, and cumulative variance.

|  | PC1 | PC2 | PC3 | PC4 | PC5 |
| --- | --- | --- | --- | --- | --- |
| Eigenvalue | 3.1815 | 2.383 | 1.548 | 0.711 | 0.525 |
| Variance (%) | 53.4% | 2909% | 12.6% | 2.7% | 1.5% |
| Cumulative Variance (%) | 53.4% | 83.3% | 95.9% | 98.5% | 100.0% |

**Supplementary Table S13:** Contributions (loadings) of the altitude and the 19 bioclimatic variables of the principal Components (PCs).

|  | PC1 | PC2 | PC3 | PC4 | PC5 |
| --- | --- | --- | --- | --- | --- |
| BIO1 | 8.27 | 2.30 | 0.83 | 1.46 | 1.83 |
| BIO2 | 5.67 | 2.55 | 8.90 | 13.17 | 0.45 |
| BIO3 | 0.46 | 3.18 | 25.94 | 12.38 | 31.68 |
| BIO4 | 5.88 | 6.47 | 0.04 | 1.94 | 8.99 |
| BIO5 | 1.22 | 11.91 | 6.78 | 6.58 | 0.76 |
| BIO6 | 9.75 | 0.01 | 0.03 | 0.86 | 2.68 |
| BIO7 | 6.03 | 5.16 | 2.08 | 6.40 | 4.81 |
| BIO8 | 1.13 | 8.32 | 13.96 | 8.68 | 11.72 |
| BIO9 | 4.84 | 4.44 | 9.54 | 5.60 | 0.00 |
| BIO10 | 4.91 | 8.32 | 1.15 | 0.25 | 0.08 |
| BIO11 | 9.46 | 0.13 | 0.71 | 1.25 | 4.13 |
| BIO12 | 0.01 | 13.23 | 9.50 | 3.44 | 0.06 |
| BIO13 | 3.47 | 9.83 | 1.96 | 2.74 | 9.87 |
| BIO14 | 7.00 | 1.82 | 5.21 | 12.17 | 0.41 |
| BIO15 | 8.22 | 1.65 | 1.11 | 8.66 | 1.19 |
| BIO16 | 2.82 | 11.28 | 2.14 | 0.61 | 6.04 |
| BIO17 | 6.78 | 1.56 | 7.63 | 8.11 | 0.24 |
| BIO18 | 8.26 | 2.81 | 0.03 | 0.43 | 0.12 |
| BIO19 | 5.81 | 5.02 | 2.42 | 5.27 | 14.93 |

**Supplementary Table S14:** Model selection of egg to adult emergence rate, of different *Ceratitis capitata* populations, in two different overwintering hosts (apples and bitter oranges) based on Bayesian Information Criterion (BIC). The model selection included Latitude, PC1 and PC2, host and treatment as interaction terms.

| Number of variables in the model | Best model | Adj. R2 | AIC | BIC |
| --- | --- | --- | --- | --- |
| 1 | Treatment | 0.2712 | 7556.348 | 7567.332 |
| 2 | Treatment + Host | 0.3245 | 7420.961 | 7437.437 |
| 3 | Latitude + Treatment + Host | 0.3617 | 7320.349 | 7342.318 |
| 4 | Latitude + PC1 + Treatment + Host | 0.3645 | 7313.533 | 7340.994 |
| 5 | Latitude + PC1 + PC2 + Treatment + Host | 0.3719 | 7293.463 | 7326.416 |
| 6 | Latitude + PC1 + PC2 + Treatment + Host + PC2*Host | 0.4369 | 12191.8188 | 12235.7565 |
| 7 | Latitude + PC1 + PC2 + Treatment + Host + PC2*Host + Treatment*Host | 0.4518 | 12144.6456 | 12194.0754 |
| 8 | Latitude + PC1 + PC2 + Treatment + Host + Latitude*Host + PC1*Host + PC2*Host | 0.5116 | 11938.3119 | 11993.2339 |
| 9 | Latitude + PC1 + PC2 + Treatment + Host + Latitude*Host + PC1*Host + PC2*Host + Treatment*Host | 0.5344 | 11853.6547 | 11914.0689 |
| 10 | Latitude + PC1 + PC2 + Treatment + Host + Latitude*Host + PC1*Host + PC2*Treatment + PC2*Host + Treatment*Host | 0.5492 | 11796.7166 | 11862.6231 |
| 11 | Latitude + PC1 + PC2 + Treatment + Host + Latitude*Treatment + Latitude*Host + PC1*Treatment + PC1*Host + PC2*Treatment + PC2*Host | 0.5591 | 11757.9647 | 11829.3634 |
| 12 | Latitude + PC1 + PC2 + Treatment + Host + Latitude*Treatment + Latitude*Host + PC1*Treatment + PC1*Host + PC2*Treatment + PC2*Host + Treatment*Host | 0.5934 | 11613.5841 | 11690.4749 |
| 13 | Latitude + PC1 + PC2 + Treatment + Host + Latitude*PC1 + Latitude*Treatment + Latitude*Host + PC1*Treatment + PC1*Host + PC2*Treatment + PC2*Host + Treatment*Host | 0.5978 | 11594.8098 | 11677.1929 |
| 14 | Latitude + PC1 + PC2 + Treatment + Host + Latitude*PC1 + Latitude*PC2 + Latitude*Treatment + Latitude*Host + PC1*Treatment + PC1*Host + PC2*Treatment + PC2*Host + Treatment*Host | 0.6035 | 11570.5632 | 11658.4385 |

**Supplementary Table S15:** Model selection of egg to adult developmental duration, of different *Ceratitis capitata* populations, in two different overwintering hosts (apples and bitter oranges) based on Bayesian Information Criterion (BIC). The model selection included Latitude, PC1 and PC2, host and treatment as interaction terms.

| Number of variables in the model | Best model | Adj. R2 | AIC | BIC |
| --- | --- | --- | --- | --- |
| 1 | Treatment | 0.7810 | 7527.444 | 7538.428 |
| 2 | Latitude + Treatment | 0.7914 | 7441.697 | 7458.174 |
| 3 | Latitude + Treatment + Host | 0.7975 | 7389.203 | 7411.172 |
| 4 | PC1 + PC2 + Treatment + Host | 0.8039 | 7332.180 | 7359.641 |
| 5 | Latitude + PC1 + PC2 + Treatment + Host | 0.8069 | 7305.177 | 7338.130 |
| 6 | Latitude + PC1 + PC2 + Treatment + Host + Latitude*Treatment | 0.8151 | 12322.4907 | 12366.4283 |
| 7 | Latitude + PC1 + PC2 + Treatment + Host + PC1*Treatment + PC2*Treatment | 0.8191 | 12283.7726 | 12333.2025 |
| 8 | Latitude + PC1 + PC2 + Treatment + Host + PC1*Treatment + PC2*Treatment + PC2*Host | 0.8217 | 12259.0377 | 12313.9597 |
| 9 | Latitude + PC1 + PC2 + Treatment + Host + Latitude*Treatment + PC1*Treatment + PC2*Treatment + PC2*Host | 0.8226 | 12251.2091 | 12311.6233 |
| 10 | Latitude + PC1 + PC2 + Treatment + Host + Latitude*Treatment + PC1*Treatment + PC2*Treatment + PC2*Host + Treatment*Host | 0.8237 | 12241.3488 | 12307.2552 |
| 11 | Latitude + PC1 + PC2 + Treatment + Host + Latitude*PC2 + Latitude*Treatment + PC1*Treatment + PC2*Treatment + PC2*Host + Treatment*Host | 0.8241 | 12237.9144 | 12309.3131 |
| 12 | Latitude + PC1 + PC2 + Treatment + Host + Latitude*PC2 + Latitude*Treatment + Latitude*Host + PC1*Treatment + PC1*Host + PC2*Treatment + Treatment*Host | 0.8242 | 12238.2124 | 12315.1033 |
| 13 | Latitude + PC1 + PC2 + Treatment + Host + Latitude*PC2 + Latitude*Treatment + Latitude*Host + PC1*Treatment + PC1*Host + PC2*Treatment + PC2*Host + Treatment*Host | 0.8241 | 12239.4700 | 12321.8531 |
| 14 | Latitude + PC1 + PC2 + Treatment + Host + Latitude*PC1 + Latitude*PC2 + Latitude*Treatment + Latitude*Host + PC1*Treatment + PC1*Host + PC2*Treatment + PC2*Host + Treatment*Host | 0.8240 | 12241.3743 | 12329.2496 |


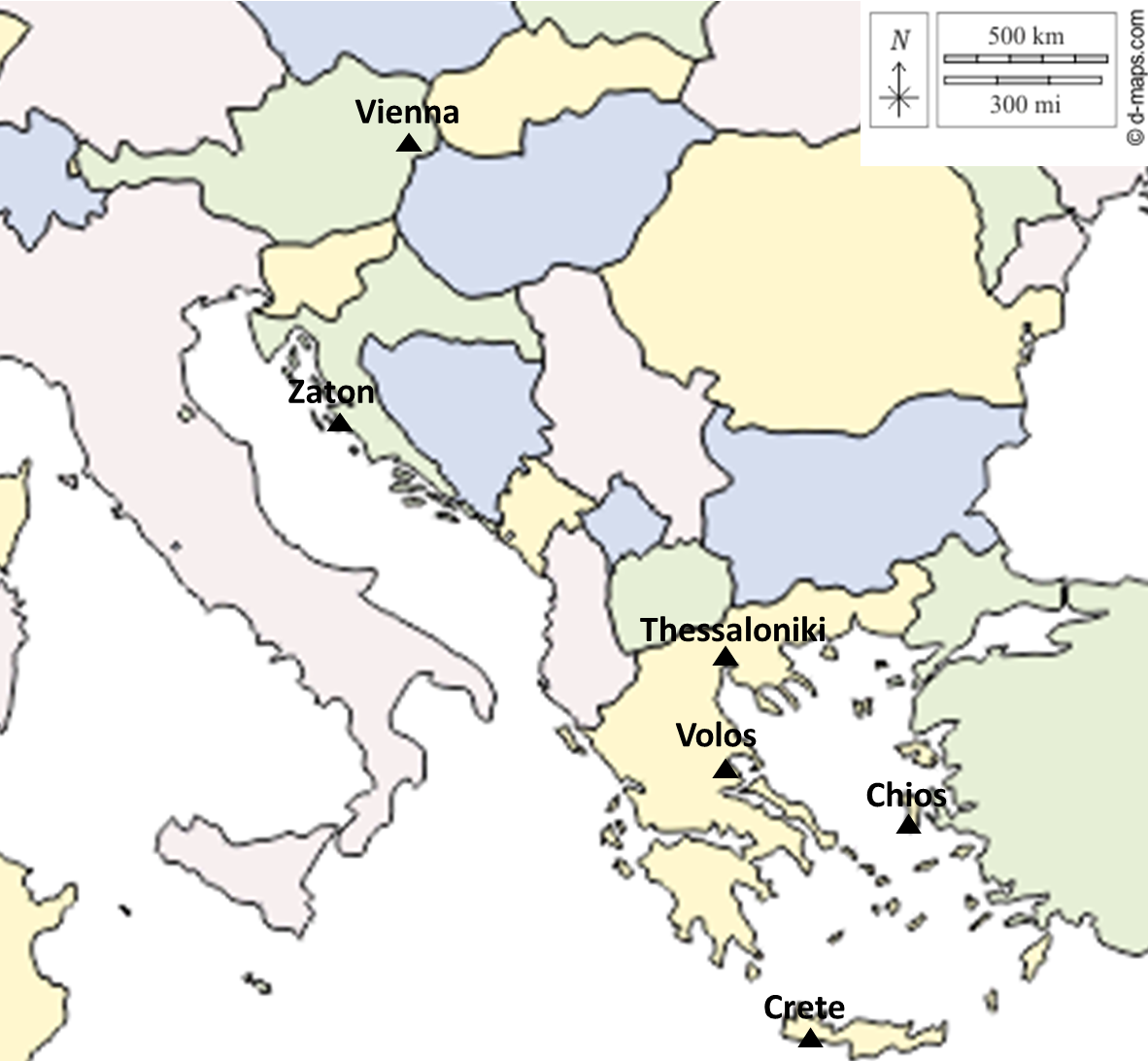


**Supplementary Figure S4:** Map with the six collection sites of *Ceratitis capitata* populations used in the current study. The map template obtained from <https://d-maps.com>.
